# Supplementary material for: Potential of PEDOT:PSS as a hole selective front contact for silicon heterojunction solar cells
Source: Sci Rep. 2017 May 19;7:2170. doi: 10.1038/s41598-017-01946-3 (PMC5438391; doi:10.1038/s41598-017-01946-3)
Supplement: Supplementary file 1 — Supplementary Information [file 41598_2017_1946_MOESM1_ESM.pdf]

Supplementary Information

Potential of PEDOT:PSS as a hole selective front contact for silicon heterojunction solar cells

*Sara Jäckle[1,2+], Martin Liebhaber[3+], Clemens Gersmann[3], Mathias Mews[4], Klaus Jäger[5], Silke Christiansen[1,2,6], Klaus Lips[3,6]**

S1 Comparison of PH1000 and F HC (Clevios, Heraeus)

For the standard preparation of PEDOT:PSS/c-Si solar cells with a metal back contact we used the polymer solution PH1000 (Heraeus Clevios) mixed with 5 vol% dimethyl sulfoxide (DMSO) and 0.1 vol% of wetting agent (FS31, Capstone), following our previous work.[1,2] The high efficiency PEDOT:PSS/c-Si/a-Si devices were fabricated with the premixed polymer formulation F HC (Heraeus Clevios). While the thickness of the polymer layers

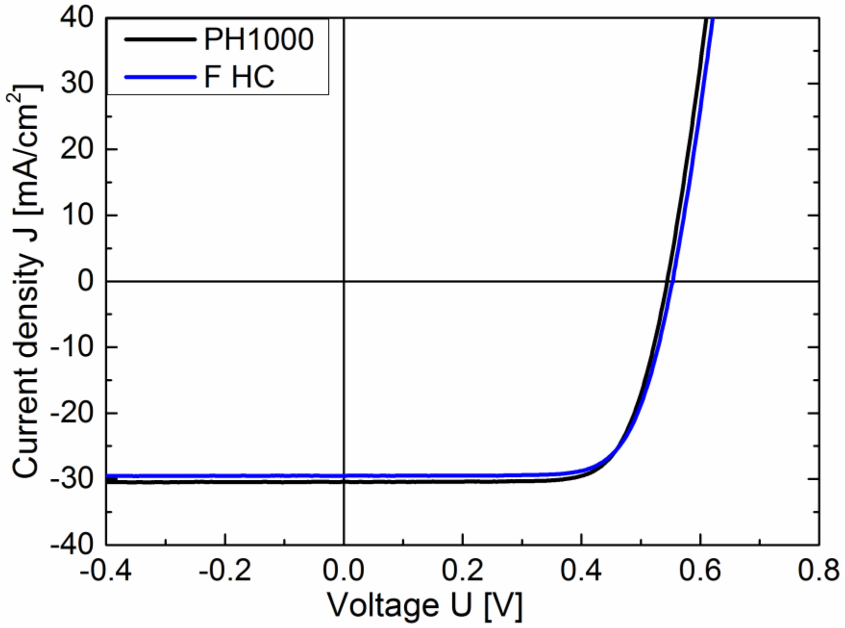

Figure S1. Photovoltaic response of silicon solar cells with a PEDOT:PSS front junction and a metal back contact for different PEDOT:PSS solutions. Extracted solar cell parameters are collected in Table S1.

	Films (on glass)		Solar cell parameters				
	d (2k) [nm]	σ [S/cm]	d [nm]	V_{oc} [V]	J_{sc} [mA/cm²]	FF	PCE [%]
**PH1000**	~125	~630	~85 (4k)	0.544	30.5	0.73	12.2
**F HC**	~70	~650	~82 (2k)	0.554	29.5	0.73	11.9

Table S1. Summary of polymer film and solar cell parameters for different PEDOT:PSS formulations (all abbreviations are defined in the text). Polymer films on glass are spin coated at 2000 rpm, while for solar cell fabrication PH1000 is spin coated at 4000 rpm and FHC at 2000 rpm.

depends on the spin coating parameter as well as to a small extend on the substrate size, under similar preparation conditions F HC leads to thinner films than PH1000 (determined by AFM measurements). The specific conductivity of both solutions, measured by the Van de Pauw method on polymer films spin coated on a glass substrate and collected in Table S1, is almost the same. To achieve a comparable polymer film thickness on silicon substrates for solar cells, PH1000 is spin coated at 4000 rpm and F HC at 2000 rpm. Figure S1 shows the photovoltaic response of solar cells fabricated on bulk-limited silicon wafers with metal back contacts featuring the two PEDOT:PSS solutions. The corresponding solar cell parameters are collected in Table S1. Besides a slight decrease in J_{SC}, which might be due to a less favorable antireflective behavior of the slightly thinner F HC layer (see also Fig. 4a in the main manuscript), the performance of the solar cell does not differ from the one prepared with PH1000. This shows that these solutions can be used interchangeable when controlling the film thickness.

S2 Optical constants of PEDOT:PSS

The optical constants of the used PEDOT:PSS formulations PH1000 and F HC were derived by spectroscopic ellipsometry (UVISEL, Horiba Jobin Yvon). For the measurements the polymer films were deposited on silicon substrates. Measurements were performed within a photon energy range from 0.6 eV - 4.8 eV and steps of 0.02 eV under an angle of incidence of

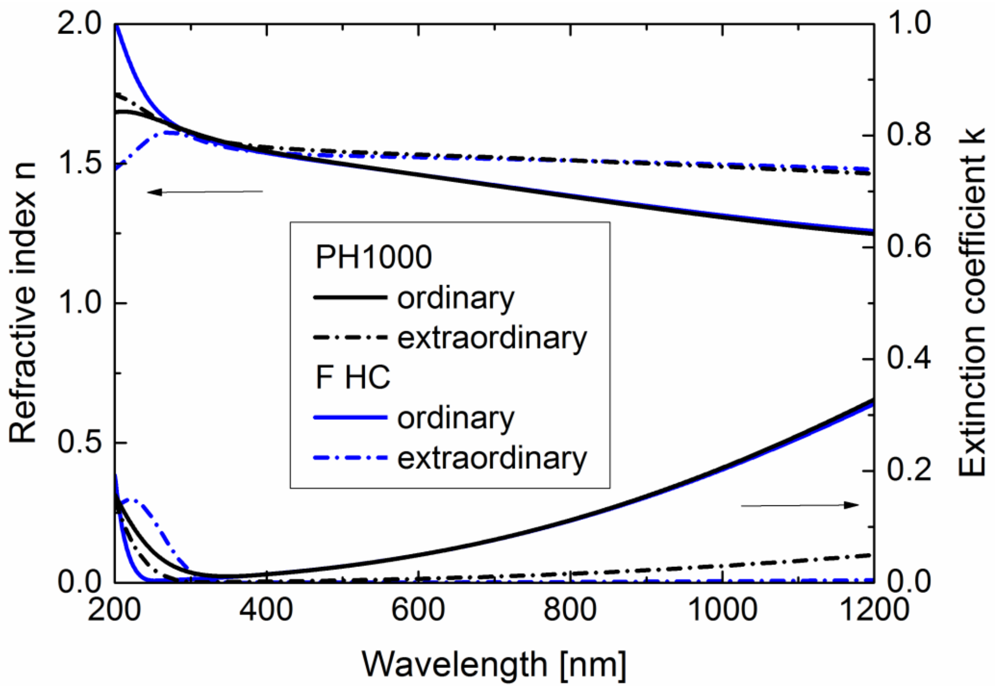

Figure S2. Optical constants of uniaxial anisotropic PEDOT:PSS measured by ellipsometry for different PEDOT:PSS formulations.

70°. The data was analyzed assuming uniaxial anisotropy of PEDOT:PSS, following Pettersson et al.[3] For each component a model consisting of a Lorentz oscillator and a Drude term, accounting for free carrier absorption in the highly doped polymer film, was used.[4] The extracted optical constants in the visible spectrum for the ordinary and extraordinary component are shown in Figure S2.

References

(1) Jäckle, S.; Mattiza, M.; Liebhaber, M.; Brönstrup, G.; Rommel, M.; Lips, K.; Christiansen, S. Junction Formation and Current Transport Mechanisms in Hybrid N-Si/PEDOT:PSS Solar Cells. *Sci. Rep.* **2015**, *5*, 13008.

(2) Pietsch, M.; Jäckle, S.; Christiansen, S. Interface Investigation of Planar Hybrid N-Si/PEDOT:PSS Solar Cells with Open Circuit Voltages up to 645 mV and Efficiencies of 12.6 %. *Appl. Phys. A* **2014**, *115* (4), 1109–1113.

(3) Pettersson, L. A. A.; Carlsson, F.; Inganäs, O.; Arwin, H. Spectroscopic Ellipsometry Studies of the Optical Properties of Doped poly(3,4-Ethylenedioxythiophene): An Anisotropic Metal. *Thin Solid Films* **1998**, *313-314*, 356–361.

(4) Liu, Q.; Imamura, T.; Hiate, T.; Khatri, I.; Tang, Z.; Ishikawa, R.; Ueno, K.; Shirai, H. Optical Anisotropy in Solvent-Modified poly(3,4-Ethylenedioxythiophene): Poly(styrenesulfonic Acid) and Its Effect on the Photovoltaic Performance of Crystalline Silicon/organic Heterojunction Solar Cells. *Appl. Phys. Lett.* **2013**, *102* (24).
